# Epidemiological and Virological Characterizations of the 2014 Dengue Outbreak in Guangzhou, China

**DOI:** 10.1371/journal.pone.0156548

**Published:** 2016-06-03

**Authors:** Hui Zhao, Fu-Chun Zhang, Qin Zhu, Jian Wang, Wen-Xin Hong, Ling-Zhai Zhao, Yong-Qiang Deng, Shuang Qiu, Yu Zhang, Wei-Ping Cai, Wu-Chun Cao, Cheng-Feng Qin

**Affiliations:** 1 Department of Virology, State Key Laboratory of Pathogen and Biosecurity, Beijing Institute of Microbiology and Epidemiology, Beijing, China; 2 Guangzhou Eighth People’s Hospital, Guangzhou Medical University, Guangzhou, China; Wuhan University, CHINA

## Abstract

Dengue used to be recognized as an imported and sporadic disease in China. Since June 2014, an unexpected large dengue outbreak has attacked Guangzhou, China, resulting in more than 40,000 cases. Among the 1,942 laboratory-confirmed hospitalized dengue cases, 121 were diagnosed as severe dengue according to the 2009 WHO guideline, and 2 patients finally died. Laboratory diagnosis and virus isolation demonstrated that the majority (96%) cases were caused by dengue virus serotype 1 (DENV-1), and the others by serotype 2 (DENV-2). 14 DENV strains were isolated from the sera of acute-phase dengue patients during this outbreak, and the complete envelope (E) gene of 12 DENV-1 strains and two DENV-2 strains were determined using RT-PCR assay. Phylogenetic analysis based on the E gene revealed the DENV-1 strains isolated during the outbreak belonged to genotype I and V, respectively. These isolates formed three clades. DENV-2 isolates were assigned to the same clade belonging to genotype cosmopolitan. These strains isolated in 2014 were closely related to the isolates obtained from the same province, Guangdong, in 2013. No amino acid mutations known to increase virulence were identified throughout the E protein of isolates in 2014. These results indicate that dengue is turning into endemic in Guangdong, China, and extensive seroepidemiological investigation and mosquito control measures are critically needed in the future.

## Introduction

Today, dengue is well known as the most prevalent and worldwide expansive mosquito-borne viral disease of human beings, with an increasing frequency and magnitude of epidemics and severe disease. Recent computer analysis suggested that about 390 million infections occur annually in more than 100 countries [1], most of those are tropical and subtropical areas. In recent years, increased outbreaks of dengue have been found in previously unaffected areas including Europe, America and Japan [2–4], as a result of a combination of factors, including global climate change, rapid urbanization, international travel and trade, or viral evolution and adaption. The continuing circulation and spread of dengue poses huge public health and economic burden especially in developing countries of the South-East Asia Region and Western Pacific Region [1, 5].

Dengue has been reported in China for a long time, however, the disease burden and transmission risk remains largely unknown. During the past decades, several sporadic epidemics have been reported in several provinces in Southern China [6, 7]. Previous epidemiological and phylogenetic analysis has deduced all these autochthonous epidemics are caused by imported cases from South-East Asia [8, 9]. Guangzhou city is the largest metropolis in southern China with a population of over 13 million. Guangzhou has a typical subtropical monsoon climate with hot-humid summer and mild-dry winter. *Aedes albopictus*, that can survive the winter, is believed to be the only transmission vector of dengue in Guangzhou. For the past decades, sporadic epidemics were recorded in Guangzhou every year, and all four serotypes of dengue (DENV-1 to DENV-4) have been detected, although the DENV-1 genotype I was still predominant [10–12]. All the patients reported mild symptoms and few severe diseases or mortality have been ever recorded.

On 28 June 2014, an 80-year-old male patient admitted to Guangzhou Eighth People’s Hospital due to high fever, headache and myalgia, and he was laboratory diagnosed as autochthonous dengue. Subsequently, an unexpected dengue outbreak attacked Guangzhou, resulting in 43,031 laboratory-confirmed dengue cases as of Oct, 2014. This outbreak is the largest and most severe epidemic of dengue fever documented in China [6], and we reported the main epidemiological and virological features of this outbreak in this study.

## Materials and Methods

### Ethics statement

All the adult participants provided written informed consent, and parents/guardians provided informed consent on behalf of minors included in the study. The study was approved by the ethical committee of the Guangzhou Eighth People’s Hospital (Reference Number 20100835) according to the medical research regulations of China.

### Study design

This is an epidemiological and virological descriptive study. A total of 1,942 laboratory-confirmed patients with dengue hospitalized in Guangzhou Eighth People’s Hospital from August to October 2014 were enrolled in this study. Patients were classified into dengue and severe dengue according to the 2009 WHO guidelines [13]. Patients’ demographic, epidemiological data, laboratory data, co-morbidity conditions and treatment regimens were immediately retrieved from medical records.

### Laboratory diagnosis

All cases were confirmed according to the 2009 WHO guidelines associated with detection of nonstructural protein 1 (NS1) and/or viral RNA on patients’ serum. Serum sample were collected on admission. Viral RNA was directly extracted from the serum sample using a QIAamp Viral RNA Mini Kit (Qiagen, Valencia, CA, USA) according to the manufacturer’s instructions. Dengue genome was detected using real-time RT-PCR assay (DAAN, China) to determine specific serotype. Dengue NS1 antigen was determined in acute-phase serum samples by enzyme immunoassay (Diagnostic Kit for Dengue Virus NS1 Antigen, WANTAI, China). Serum IgG and IgM antibodies in acute-phase serum samples against DENV were detected by Dengue IgM and IgG capture ELISA (PanBio, Australia) according to the recommended protocol. The secondary infections distinguished from primary were defined as IgG positivity during the acute phase [14].

### Virus isolation and genome sequencing

Acute phase sera from 44 patients diagnosed positive with viral genomic RNA were inoculated in C6/36 mosquito cells obtained from ATCC for virus isolation as previously described [15]. When complete cytopathic effects (CPE) were observed, culture supernatants were collected for genome sequencing. In brief, viral RNA was extracted from the supernatant of the virus-infected C6/36 cell culture using a QIAamp Viral RNA Mini Kit. To amplify E gene, RT-PCR was performed using a SuperScript III One-Step RT-PCR System and the primers for E gene. The following two oligonucleotide primers 5′-GCA CAT GCC ATA GGA ACA TCC-3′ and 5′-CTC TCA CCA AAA GGC GGT TCT-3′, and 5′-AGG AAC AGA TGC ACC ATG CAA-3′ and 5′-ATC TTT CCT GTG ACT GTT GTG-3′ were used to amplify complete E gene of DENV-1. For complete E gene of DENV-2, two primer pairs 5′-CAA ACA CCC CGC CAC TCT AAG GA-3′ and 5′-ACA ATG TCA CGA CCC CCA CTA ATA-3′, and 5′-CCC CAT GCA AAG AAA CAG GAT-3′ and 5′-TTG AGG CCG CAG GGA ACG-3′ were used.

### Phylogenetic analysis

Newly isolated DENV E gene sequences, as well as those available from the GenBank were aligned by using MUSCLE [16]. Phylogenetic trees were inferred using the Metropolis-coupled Markov Chain Monte Carlo (MCMCMC) method in MrBayes v3.1.2 [17], and the GTR+I+Г_4_ model was selected for MrBayes analyses.

### Statistical analysis

The OpenEpi version 2.3.1 software was used to analyze the difference between the two rates. Fisher exact test was performed for statistical comparison. Differences were considered significant at p<0.05.

## Results

### Characteristics of the study population

**Fig 1:**
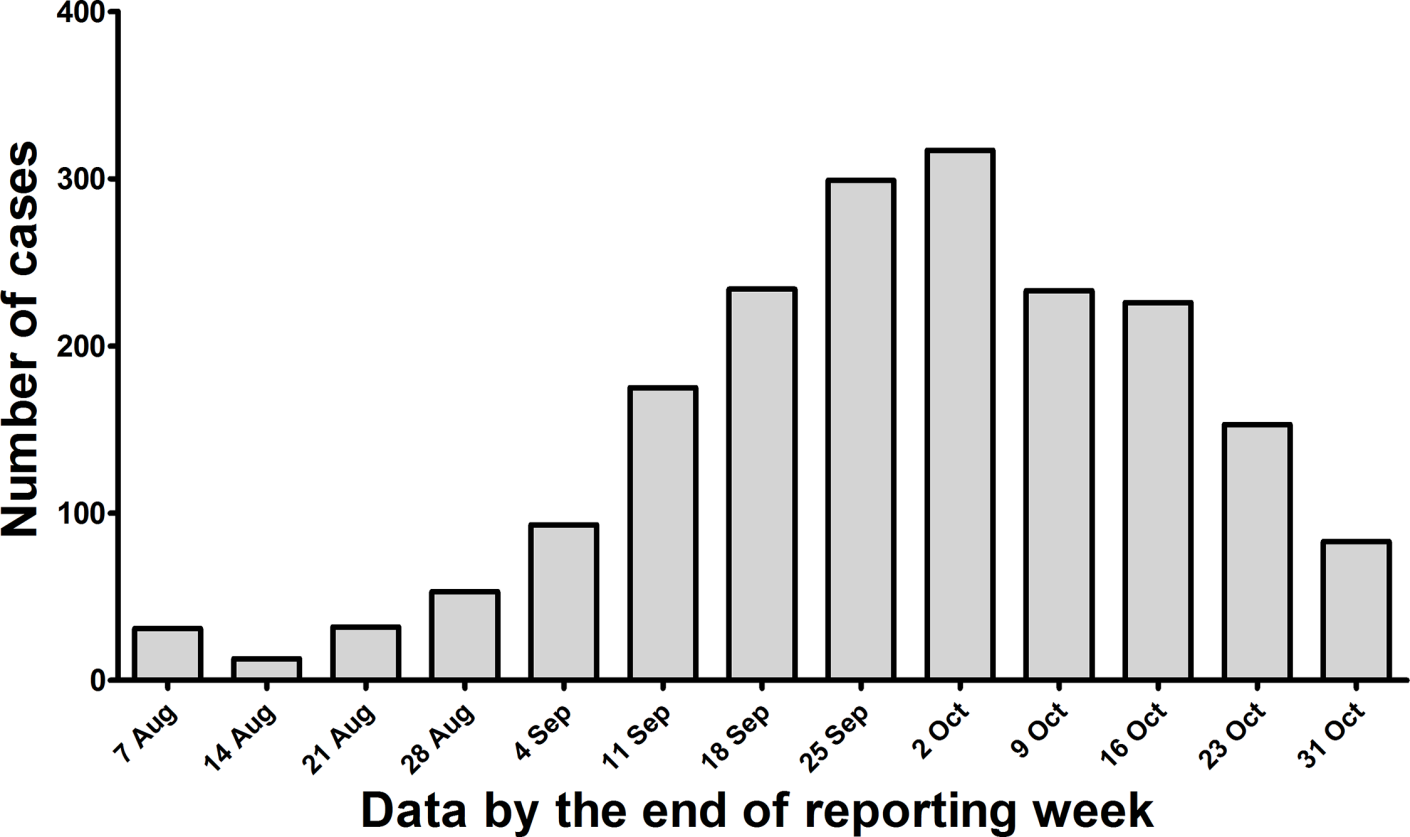
Number of the hospitalized dengue cases, Guangdong province, from 1 August to 31 October, 2014.

This report describes 1,942 hospitalized cases that had been laboratory diagnosed according to the diagnosis and treatment guideline for dengue issued by Chinese CDC. Of those, 121 cases were identified as severe dengue according to the 2009 WHO guideline[13]. On average, 149 cases (range = 13–319) were hospitalized per week from 1 August to 31 October (Fig 1). All of the patients were confirmed with serologic or virological examination for DENV. Table 1 summarizes the demographic, clinical and laboratory features of the dengue cases. The median age of patients from dengue fever was 44.2 ± 21.5 years old and 58.6 ± 22.6 years old in severe dengue cases. The large majority of dengue fever cases fell between 15 and 49 years of age. However, in severe dengue cases, the most affected age group was 65 years of age or older, and no infected cases below 15 years of age were identified during this epidemic.

**Table 1:** Characteristics of hospitalized dengue cases during the outbreak.

Characteristic	Total	Dengue fever	Severe dengue	p value
**Number of cases**	1942	1821	121	
**Age** (mean ± range)	44.7±18.4	44.2±21.5	58.6±22.6	<0.001
0 to 14 years	174(8.9%)	174 (9.6%)	0 (0%)	<0.001
15 to 49 years	948 (48.8%)	904 (49.6%)	44 (36.4%)	0.0433
50 to 64 years	433 (22.3%)	417 (22.9%)	16 (13.2%)	0.0275
≧65years	387(19.9%)	326 (17.9%)	61 (50.4%)	<0.001
**Gender**				
Female	893 (46.0%)	831 (45.6%)	62 (51.2%)	0.2698
Male	1049 (54.0%)	990 (54.4%)	59(48.8%)	0.2698
Immune status*				
Primary infection	81(49.7%)	54(52.4%)	27(45%)	0.4521
Secondary infection	82 (50.3%)	49(47.6%)	33(55%)	0.4521
**Serotype**	611	546	65	
DENV-1	570 (96.0%)	513 (97.0%)	57 (87.7%)	0.6806
DENV-2	24 (4.0%)	16 (3.0%)	8 (12.3%)	0.0050

Immune status*: Primary infection: only anti-dengue IgM was positive; Secondary infection: both anti-dengue IgM and IgG were positive or only IgG was positive using acute-phase serum samples from patients.

163 acute-phase serum samples were collected for serologic examination to determine primary or secondary infection using ELISA (Panbio, Australia). Among the severe dengue patients, 45% was primary infection, and the others belonged to secondary infection. Interestingly, no significant differences in the severity of disease between primary and secondary dengue infections were observed in patients from severe dengue and dengue fever group (Table 1).

A total of 611 acute-phase serum samples were detected using real-time RT-PCR assay. As shown in Table 1, of those, 594 were positive for DENV (97.2%). Further, 570 cases were infected by DENV-1 (96%) and others by DENV-2, which demonstrated that two serotypes of DENV co-circulated during this outbreak: DENV-1 and DENV-2, with serotype 1 predominating. Among the severe dengue patients, 12% were infected with DENV-2, which was significantly higher than that in dengue fever cases (*P* = 0.005).

### Virus isolation and complete envelope gene sequences

The acute phase serum was directly inoculated into C6/36 cells to isolate the pathogen. Five days post inoculation on the third passage in the cells, CPE caused by DENV infection, characterized by swelling, aggregation and cell fusion were observed. Finally, 14 DENV strains were isolated from the sera of acute-phase dengue patients. Furthermore, the complete envelope (E) gene of 12 DENV-1 strains and two DENV-2 strains were determined using RT-PCR assay and submitted to GenBank under the Accession number KU672341 (DENV-1/GZ01), KU672340 (DENV-1/GZ02), KU672339 (DENV-1/GZ09), KP723473 (DENV-1/GZ27), KP723474 (DENV-1/GZ32), KP723475 (DENV-1/GZ33), KP723476 (DENV-1/GZ35), KP723477 (DENV-1/GZ37), KU672338 (DENV-1/GZ44), KU672337 (DENV-1/GZ48), KU672336 (DENV-1/GZ81), KU672342 (DENV-1/GZ84), KP012546 (DENV-2/GZ05), KP723478 (DENV-2/GZ25), respectively.

### Phylogenetic analysis

**Fig 2:**
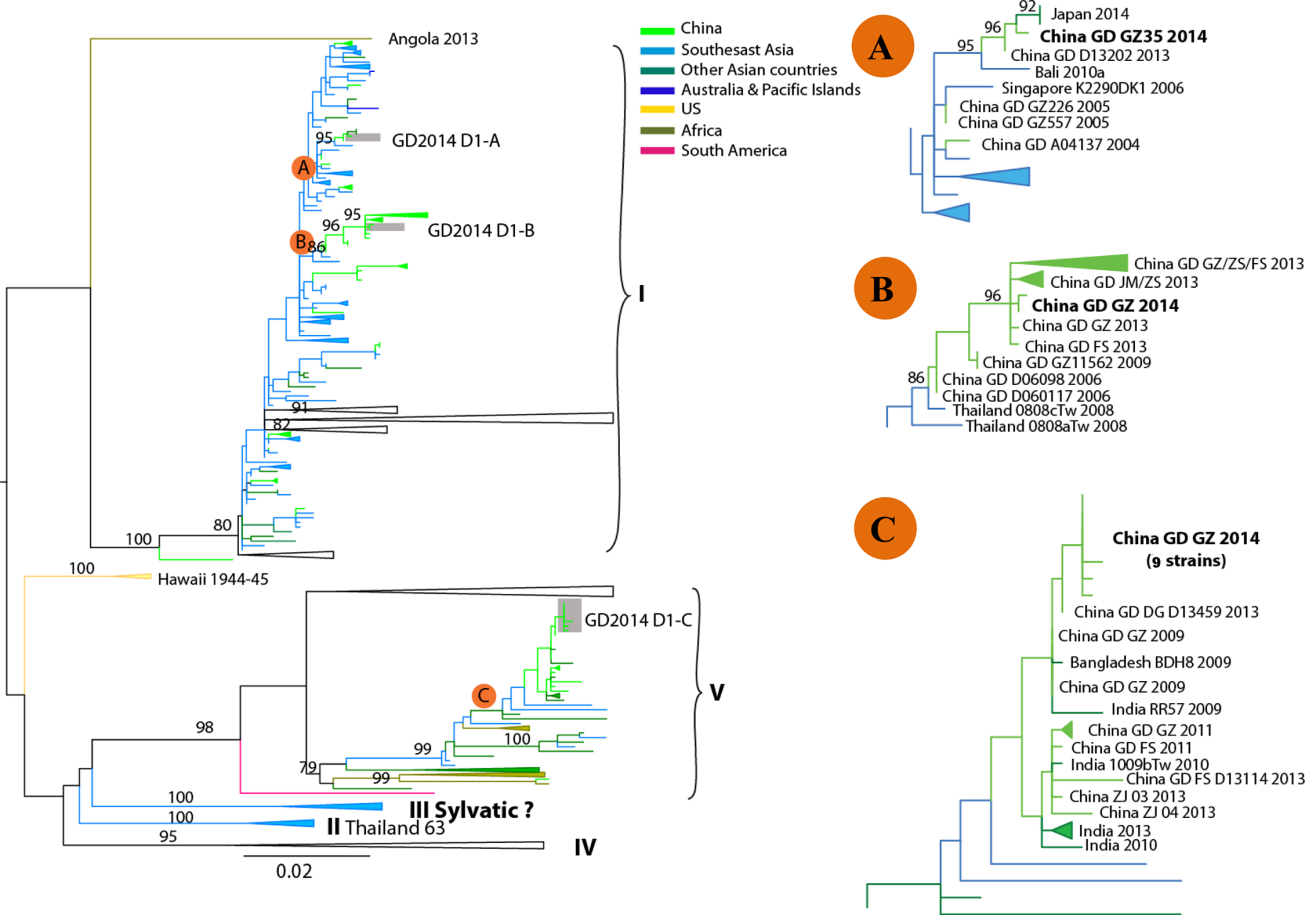
Phylogenetic tree of dengue virus serotype 1 (DENV-1) sequences from Guangzhou compared with other DENV-1sequences deposited in GenBank. The tree was constructed on the basis of complete sequence of the E gene. Sequences from different geographic regions are shown by different colors. For clarity, the GD2014 D1-A, B and C epidemic clade is shown enlarged in the inset at right. Scale bar indicates nucleotide substitutions per site. Strains are labeled as follows: location strain name date (year) of collection. China GD GZ 2014 of the B clade represent a group of the newly obtained sequences from this outbreak, including GZ32 and GZ84 strains. China GD GZ 2014 of the C clade represent GZ01, GZ02, GZ09, GZ27, GZ33, GZ37, GZ44, GZ48 and GZ81 strains from this outbreak. Abbreviations: GD (Guangdong province); GZ (Guangzhou city); ZS (Zhongshan city); FS(Foshan city); JM (Jiangmen city).

The phylogenetic analysis was carried out based on the complete E gene. As shown in Fig 2, the data suggested that DENV-1 strains isolated during the outbreak belonged to genotype I and V, respectively. These isolates formed three clades (GD2014 D1-A, B and C). GD2014 D1-A (GZ35 strain) was closely related to the isolate (GD D13202) obtained from the same province, Guangdong in 2013, and highly similar to isolates obtained in Japan (2014), Bali (2010) and Singapore (2006). GD2014 D1-B (GZ32 and GZ84 strain) was almost identical to those strains obtained in Guangdong from 2006 to 2013, and clustered together with isolates obtained in Thailand (2008). The most recent origins of GD2014 D1-C (GZ01, GZ02, GZ09, GZ27, GZ33, GZ37, GZ44, GZ48 and GZ81 strain) were isolates obtained from Guangdong in 2013 (DG D13459) and 2009, and showed high similarity with isolates obtained from Bangladesh and India in 2009.

**Fig 3:**
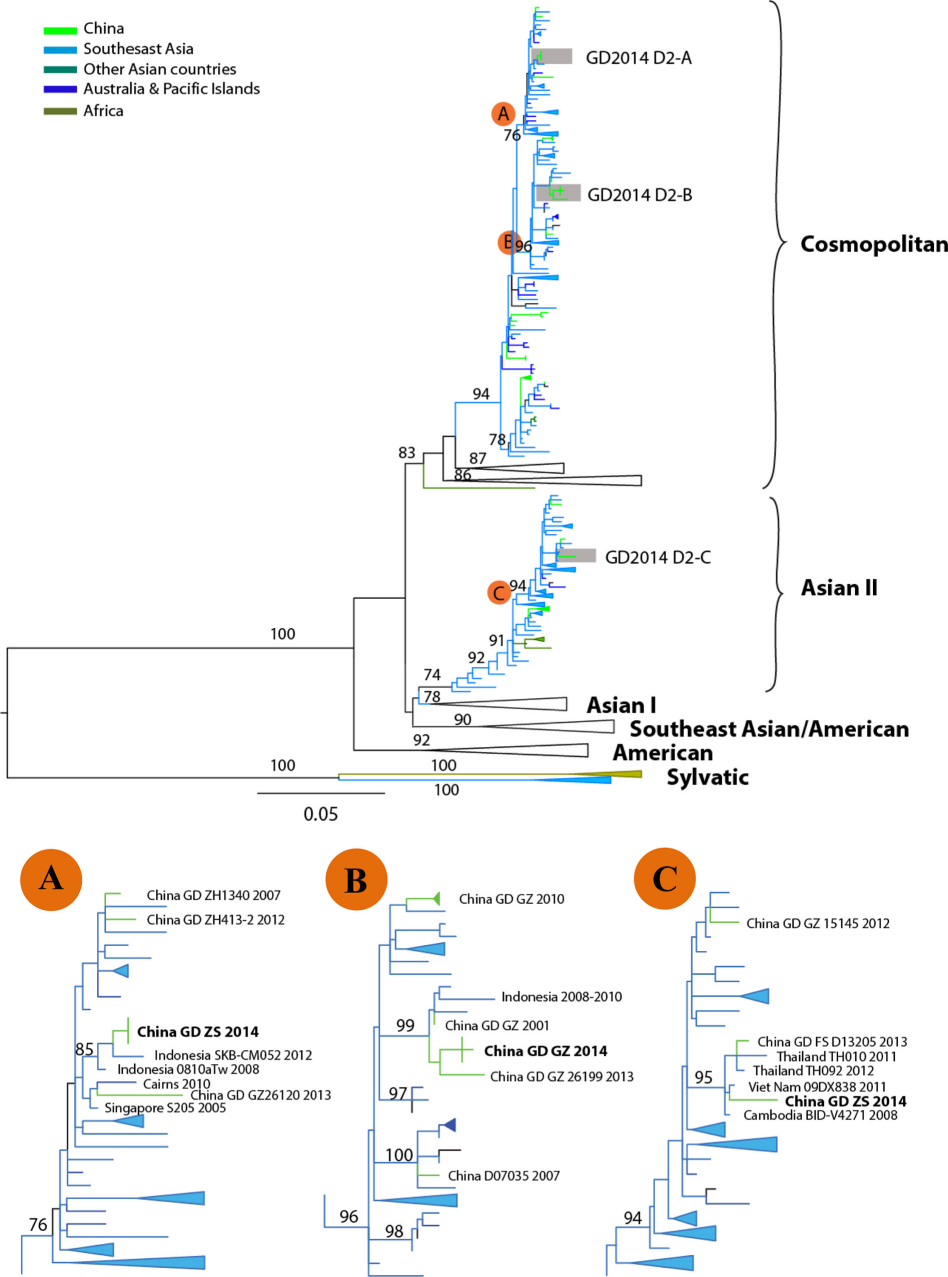
Phylogenetic tree of dengue virus serotype 2 (DENV-2) sequences from Guangzhou compared with other DENV-2 sequences deposited in GenBank. The tree was constructed on the basis of complete sequence of the E gene. Sequences from different geographic areas are shown by different colors. For clarity, the GD2014 D2-A, B and C epidemic clade is shown enlarged in the inset at lower. Scale bar indicates nucleotide substitutions per site. Strains are labeled as follows: location strain name date (year) of collection. China GD GZ 2014 represent the newly obtained sequences (GZ05 and GZ25 strains) from this outbreak. Abbreviations: GD (Guangdong province); GZ (Guangzhou city); ZS (Zhongshan city); FS (Foshan city); JM (Jiangmen city).

Phylogenetic analysis of DENV-2 strains isolated during the outbreak showed that these two newly isolated strains (GZ05 and GZ25) were assigned to the same clade (GD2014 D2-B) belonging to genotype cosmopolitan (Fig 3), which was closely related to the strain (GZ26199) isolated from Guangdong in 2013, and shared the same origins of Indonesia from 2008 to 2010. Other Guangdong strains isolated in 2001, 2007 and 2010, were also clustered into this clade. In addition, some strains isolated from other laboratory during this outbreak were assigned to the clade GD2014 D2-A and GD2014 D2-C. Zhongshan strains (GD ZS 2014) situated in these two clades were highly similar to isolates obtained in Southeast Asia (Indonesia, Singapore, Thailand, Viet Nam and Cambodia) from 2005 to 2012, along with the stains from Guangdong province (GZ26120 and FS D13205) in 2013. Together, these results suggested that multiple sources of DENV-1 and DENV-2 strains were responsible for this severe outbreak, and most stains have circulated in Guangdong for a long time.

### Amino acid mutations in the E protein of DENV-1 genotype 1 isolates in Guangdong province from 2001 to 2014

To identify whether there is any potential amino acid variation that would increase the virulence of DENV-1 genotype 1 isolates in the 2014 outbreak, the complete E gene of the isolates in 2014 was compared with other DENV-1 strains isolated in Guangdong, China, from 2001 to 2014. As shown in Table 2, all the amino acids retained identical among the strains isolated in 2004, 2006, 2009, 2013 and 2014, which was in agreement with the phylogenetic analysis results. Only 1 nonsynonymous mutation was identified in five isolates, and three nonsynonymous mutations were identified in two isolates. However, the biological effects of these mutations remain unknown. Current sequence analysis failed to identify any amino acid mutations in the E protein that would make an obvious connection between viral virulence and disease severity.

**Table 2:** Summary of mutations resulting in amino acid changes in envelope (E) protein gene of DENV-1 genotype 1 isolates in Guangdong province during 2001–2014.

Isolates	Collection date	Amino acid position in the E protein								
		55	83	114	171	180	227	338	425	461
GZ35	2014	V	V	I	T	A	S	S	I	V
GZ32	2014	-	-	-	-	-	-	-	-	-
GZ84	2014	-	-	-	-	-	-	-	-	-
GZ27	2013	-	-	-	-	-	-	-	-	-
GZ/13795	2012	I	-	-	-	-	-	-	-	-
11/GZ/02	2011	I	-	-	-	-	-	-	-	-
10/GZ/11396	2010	-	-	-	-	-	-	L	-	-
GZ/09/11562	2009	-	-	-	-	-	-	-	-	-
GZ7849	2008	-	M	-	S	-	-	-	V	-
D07gz68	2007	-	-	-	-	-	T	L	-	A
GZ/XNC	2006	-	-	-	-	-	-	-	-	-
GZ557	2005	-	-	-	-	V	-	-	-	-
A04137	2004	-	-	-	-	-	-	-	-	-
GZ257	2001	-	-	L	-	-	-	-	-	-

V = valine; I = isoleucine; M = methionine; T = threonine; S = serine; A = alanine; L = leucine.

## Discussion

The 2014 dengue outbreak in Guangdong province is the largest one in China, and has raised the concern of public health authorities for the etiology of the novel DENV. However, current research has not yet explained the correlation between the scale and severity of this outbreak and genetic characterization of newly isolate [15]. Further phenotype and virulence analyses should be carried out in the future, to identify the emergence of a “new” DENV stain with increased pathogenicity or replicative fitness in native mosquitoes for transmission. In this unexpected epidemic, the majority of severe cases felled the group with 65 years of age or older. Of those, 60.3% (73/121) cases had co-morbid conditions, which was also found in the dengue epidemic with a significant proportion of severe dengue case in Yunnan province, China[18], suggesting that the host factors also might play an important role in the severity of disease, and should be further investigated.

During this outbreak, DENV-1 and DENV-2 were co-circulating, although serotype 1 was predominant with two genotypes (I and V), which increased the risk of secondary infection. In 2011, a severe dengue case due to secondary DENV-1 infection, following primary DENV-2 infection, has been reported in Guangdong province [19]. OhAinle *et al*. found that interactions between DENV immunity and viral genetics drive dengue disease outcomes [20]. DENV-1 was predominant serotype in circulation and responsible for the epidemics of dengue fever in Guangdong for decades [6]. However, the endemic nature from a monotypic to a multitypic circulation of DENV occurred in Guangdong in recent years [7]. The study on viral evolution and the effects of population dynamics on viral fitness and virulence deserves close monitoring and careful research.

It is concern whether such outbreak re-emergence in the following years. Polygenetic analysis showed that the clinical DENV E sequences during this outbreak have high homology with the recent reported isolates in Guangdong, suggesting the possibility of dengue becoming endemic in Guangdong, China, although most researches suggested that the prevalence of dengue is attributed by the imported cases in China [6, 21]. Current E gene analysis of the 2014 strains has not yet explained the scale of this dengue epidemic. Further phenotype characterization should be warranted in the future. Furthermore, the introduction of *Aedes aegypti*, more effective DENV vector and the changes of the meteorological variables in Guangdong have brought high risks for severe epidemic and endemic [18, 22]. Nevertheless, dengue is not a passing problem in China, and consistent laboratory-based national surveillance response and long-term vector control are needed in the future [23–25].
